# Ferroelectric capacitive memories: devices, arrays, and applications

**DOI:** 10.1186/s40580-024-00463-0

**Published:** 2025-01-22

**Authors:** Zuopu Zhou, Leming Jiao, Zijie Zheng, Yue Chen, Kaizhen Han, Yuye Kang, Dong Zhang, Xiaolin Wang, Qiwen Kong, Chen Sun, Jiawei Xie, Xiao Gong

**Affiliations:** Department of Electrical and Computer Engineering, National University of Singapore (NUS), Singapore, 117576 Singapore

**Keywords:** Ferroelectric capacitive memory, Ferroelectric non-volatile capacitor, Ferroelectric memcapacitor, Capacitive crossbar array, Charge-domain computing

## Abstract

Ferroelectric capacitive memories (FCMs) utilize ferroelectric polarization to modulate device capacitance for data storage, providing a new technological pathway to achieve two-terminal non-destructive-read ferroelectric memory. In contrast to the conventional resistive memories, the unique capacitive operation mechanism of FCMs transfers the memory reading and in-memory computing to charge domain, offering ultra-high energy efficiency, better compatibility to large-scale array, and negligible read disturbance. In recent years, extensive research has been conducted on FCMs. Various device designs were proposed and experimentally demonstrated with progressively enhanced performance, showing remarkable potential of the novel technology. This article summarizes several typical FCM devices by introducing their mechanisms, comparing their performance, and discussing their limitations. We further investigate the capacitive crossbar array operation and review the recent progress in the FCM integration and array-level demonstrations. In addition, we present the computing-in-memory applications of the FCMs to realize ultra-low-power machine learning acceleration for future computing systems.

## Graphical Abstract

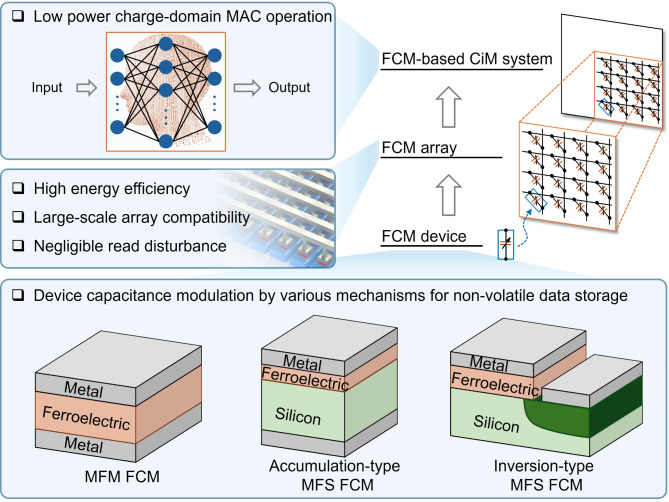

## Introduction

The increasing demand for computing power, coupled with the rapid growth in data volume and data-centric computing, is driving the search for innovative memory devices that can alleviate the “memory wall” bottleneck or even bypass it through the implementation of the compute-in-memory (CiM) paradigm [1–8]. As the device candidates for next generation massive data storage and future in-memory computing architecture, various emerging non-volatile memory (eNVM) devices have been under intensive research in the past decade [8–18]. Among them, ferroelectric memories, fueled by the development of HfO_2_-based ferroelectric materials since 2011 [19, 20], demonstrate great potential and have garnered significant interest from both academia and industry due to their low-power field-driven write mechanism, excellent complementary-metal-oxide-semiconductor (CMOS) compatibility, and scalability [21–30]. The typical ferroelectric memory devices include ferroelectric random-access memory (FeRAM) [31–35], ferroelectric tunnel junction (FTJ) [36–40], and ferroelectric field-effect transistor (FeFET) [41–50]. With the most basic metal-ferroelectric-metal (MFM) structures, FeRAM is relatively mature but suffers from destructive read operation. FTJ can be read non-destructively with junction tunnel current modulated by the polarization. However, optimization of FTJ can be extremely difficult, which requires balancing the depolarization field, tunnel current magnitude, and tunneling electroresistance ratio [36, 39]. In comparison, FeFET introduces the third terminal to decouple the memory reading and writing, exhibiting decent memory characteristics.

In addition to the three ferroelectric memory devices above, in recent years, ferroelectric capacitive memories (FCMs, also known as FeCAPs, ferroelectric NvCAPs, and ferroelectric memcapacitors) have emerged as another promising technology [51–70]. With a unique capacitive reading mechanism, the novel devices present good memory performance while maintaining the non-destructive read operation and compact two-terminal device structures.

FCMs utilize the device capacitance to represent the stored data, leading to a significant difference in the operation compared to conventional resistive memory devices such as phase change memory (PCM), resistive random-access memory (RRAM), spin-transfer-torque magnetic random-access memory (STT-MRAM), FTJ, flash (charge-trapping flash or floating-gate flash), and FeFET. These resistive memory devices modulate the conductance of the junctions or transistor channels to store data and are read by sensing the current. While such resistive devices show potential in various applications with impressive performance, several limitations originated from the current-domain operation remain unsolved, posing challenges to the implementation of these technologies in large-scale memory arrays [18, 65, 66, 71, 72].

By transferring the memory read and CiM operations to charge domain, FCMs further demonstrate several intrinsic superiorities compared to the resistive memories. First, FCMs enable the further reduction of power consumption. As capacitive memories, FCMs are able to eliminate the direct current path with ultra-high device resistance. In the write operation, write current of FCMs is significantly lower than that of the resistive devices with current-driven write mechanisms [18, 60]. In the memory read and CiM operations, FCMs can be accessed with zero-static power in principle, greatly enhancing the energy efficiency compared with resistive memories having high device conductance in the on state (low-resistance state), especially in the large-scale crossbar arrays [53, 65]. Second, with a device resistance much higher than the interconnects, FCMs effectively mitigate IR drop issue (high voltage drop along the interconnects) faced by the resistive memory devices. Third, free from IR drop issue, certain connection schemes can be implemented in the FCM crossbar array, allowing it to circumvent the sneak path problem found in resistive crossbar arrays without the need for selectors [53, 58, 59, 65, 66]. Fourth, reading of the FCMs can be performed with zero direct current (DC) voltage, guaranteeing a small voltage stress in each read operation and hence negligible disturbance to the device memory states [52–54, 57, 60–64].

This article reviews the development of various FCM devices and summarizes the recent advancements in these emerging technologies. To start with, we compare the FCM devices with MFM and metal-ferroelectric-semiconductor (MFS) structures, showing their different capacitance modulation mechanisms as well as the pros and cons. After that, we investigate the crossbar arrays based on FCMs and highlight the advantages over their resistive counterparts. Additionally, a 1-transistor-1-capacitor (1T1C) integration scheme is introduced to provide large-scale integration capabilities. Lastly, several demonstrations of the CiM applications of the FCMs are shown. By surveying the various device designs, integration methods, and applications, this review article aims to provide a comprehensive understanding of FCM technologies and present their potential to future data storage and CiM applications.

## FCM Device structures & mechanisms

In this section, we would like to introduce the FCM devices from the early explorations to the state-of-the-art demonstrations. FCM devices utilize the properties of ferroelectric materials to modulate the device capacitance, which can be realized through different mechanisms in various device structures. In this article, several mainstream FCM devices are classified into two categories according to their material stacks. The MFM FCM devices were developed based on the asymmetric capacitance versus voltage (C-V) characteristics of ferroelectric capacitor, offering the non-destructive read property for FeRAM. The MFS FCM devices utilize the semiconductor to provide larger capacitance ratio, which evolute from the accumulation-type to inversion-type to address the weak erase issue. The structures and capacitance modulation mechanisms of each device will be discussed in detail in the following subsections. Table 1 provides a summary and comparison of the various FCMs in the existing reports.

### FCMs with MFM stack

Ferroelectric capacitor with MFM stack is the key component in the FeRAM, which is the most mature ferroelectric memory with long history [73]. Thanks to the simple structure, the MFM stack is relatively easy to design and optimize, showing excellent memory performance as well as integration flexibility with BEOL compatibility. The large-scale integration of the HfO_2_-based FeRAM has been demonstrated at chip level with performance and memory density outperforming the commercialized products [31]. The stored data in FeRAM is read by sensing the transient current induced by the polarization switching of the ferroelectric layers. Since the reading process resets the memory states, the cell must be rewritten to its original state after reading, leading to additional power consumption and latency. This destructive read issue can be regarded as a main drawback of FeRAM.

**Fig. 1:**
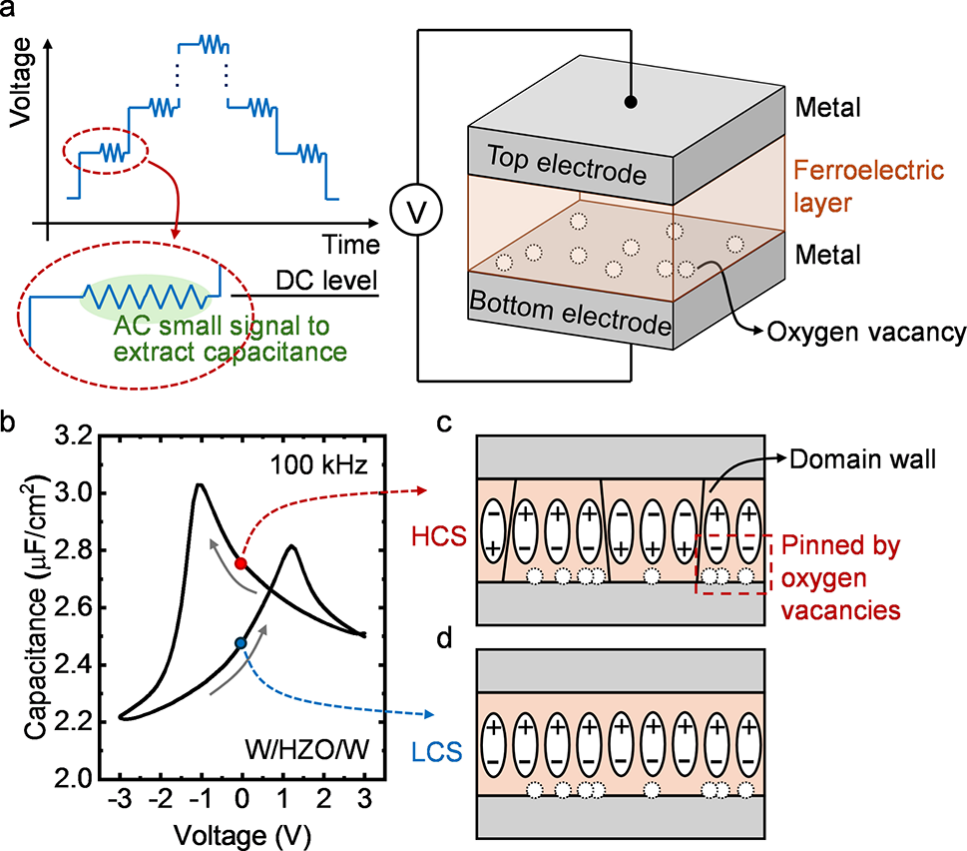
**(a)** Device structure of the MFM FCM and the C-V measurement scheme. In the voltage signal for the C-V measurement, the high frequency AC small signals are superimposed on the varying DC voltage levels to measure the device capacitance under different voltage biases. **(b)** Measured C-V curve of a MFM FCM. The top and bottom electrodes are W deposited by sputtering process. The ferroelectric layer is 10 nm 50% Zr-doped HfO_2_ (HZO) thin film grown by atomic layer deposition (ALD) at 300 °C. The AC small signal for C-V measurement has a root-mean-square amplitude of 50 mV and frequency of 100 kHz. **(c)** & **(d)** Illustration of the capacitance modulation mechanism. **(c)** In HCS, some domains are pinned by the oxygen vacancies at the interface, resulting in more domain walls compared to **(d)** LCS

Luo et al. and Hur et al. studied the asymmetric C-V characteristics of the MFM stacks and proposed using the tunable small-signal capacitance to read the devices non-destructively as FCM [52, 53]. As shown in Fig. 1 (a), the MFM FCM device features a ferroelectric layer sandwiched by two metal electrodes, identical to the ferroelectric capacitor structure in typical FeRAM. To measure the capacitance of the device under different voltage biases, a voltage signal is applied, where alternating current (AC) small signals for capacitance measurement are superimposed on the varying DC voltage levels. Ideally, for an MFM capacitor with the same metal for top and bottom electrodes, the C-V curve exhibits a symmetric butterfly shape and intersects at zero bias [51, 55, 74, 75]. However, in fabricated devices, asymmetric C-V curves are often observed [52, 53]. Figure 1 (b) displays an example of C-V curve measured in a MFM device, where, depending on the polarization of the ferroelectric layer, the device capacitance is different at zero voltage bias. The asymmetry can be explained by the oxygen vacancies at the interface introduced during the fabrication process [52, 53, 65]. As illustrated in Fig. 1 (c), under the positive voltage, some dipoles in the ferroelectric layer are pinned by the oxygen vacancies and fail to switch, leading to more domain walls at zero bias compared with the device after negative voltage [shown in Fig. 1 (d)]. The different numbers of domain walls further result in the difference in the device capacitance [52]. Therefore, two capacitance states can be observed at zero voltage bias, which are defined as high capacitance state (HCS) and low capacitance state (LCS). In addition to this process-induced non-ideal factor, the C-V asymmetry can also be realized by designing the asymmetric device structure such as introducing the interfacial layer (IL) and using different electrode materials [54, 56].

With the two separated capacitance states at zero bias, the MFM device can be operated as FCM. The data stored in the device can be read by sensing the device capacitance at zero voltage bias without disturbing the polarization state, providing a non-destructive read strategy for the conventional FeRAM. With the compact MFM structure, the FCM also enjoys the excellent integration flexibility on BEOL for high density memory and monolithic integration with front-end-of-line (FEOL) CMOS. However, as summarized in Table 1, the memory performance of the MFM FCM still needs to be optimized, especially the capacitance ratio between the HCS capacitance and LCS capacitance (*C*_HCS_/*C*_LCS_). Due to the capacitance modulation mechanism, the typical values of the MFM FCM capacitance ratio are below 1.30, which limits the sensing margin and makes it challenging to develop multi-level memory for the storage of multi-bit data in one device. Furthermore, such a low capacitance ratio may degrade the retention and endurance performance of the memory device. A slight shift in the device C-V characteristics during the data retention or repetitive write operations can further narrow the small sensing margin, potentially leading to the failure of the memory.

### FCMs with MFS Stack

In addition to the devices utilizing the intrinsic polarization-dependent permittivity of ferroelectric materials, FCMs exploiting the properties of MFS structures—or more precisely, metal-ferroelectric-insulator-semiconductor (MFIS) structures when accounting for the interfacial layer formed during the fabrication process—have also been proposed, featuring a dramatically enhanced capacitance ratio (see Table 1).

#### Accumulation-type FCM

**Fig. 2:**
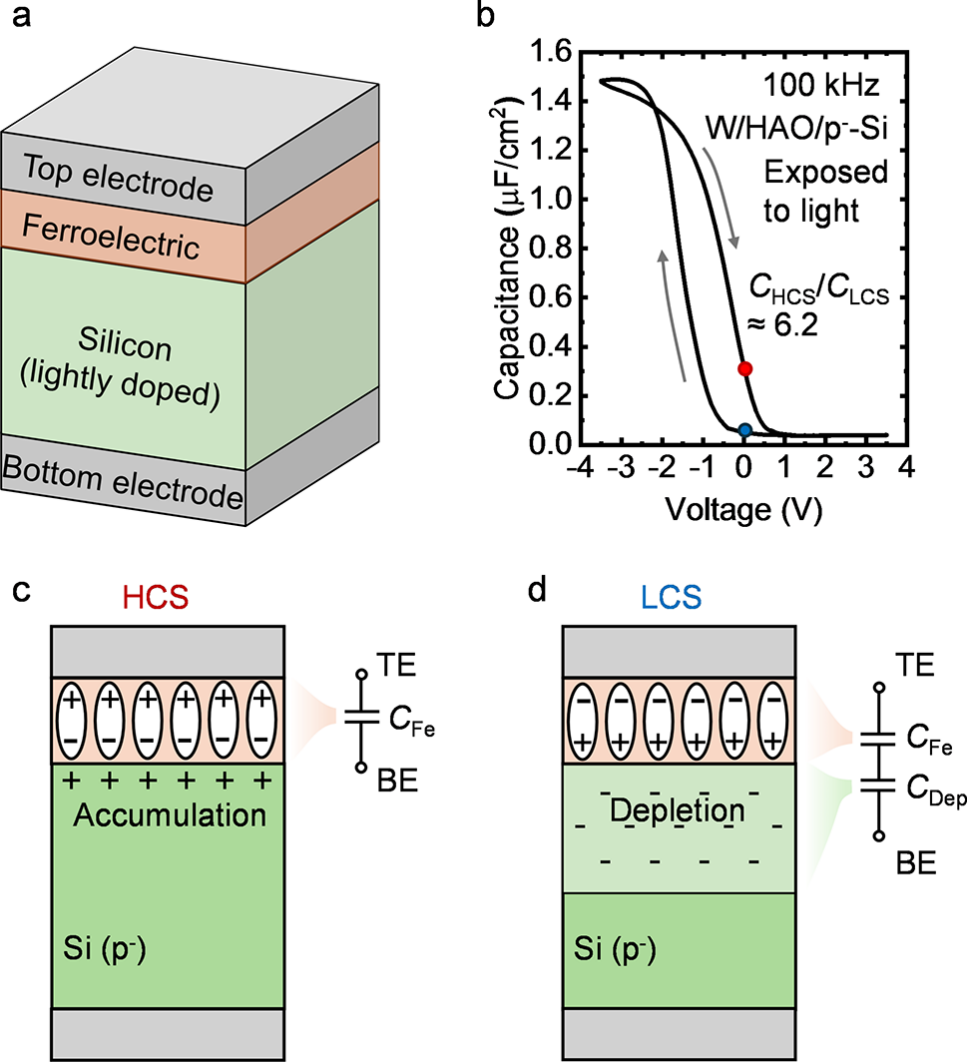
**(a)** Device structure of the accumulation-type FCM [57]. **(b)** Measured C-V curve of an accumulation-type FCM. The TE is W deposited by sputtering process. The ferroelectric layer is 10 nm HAO grown by ALD at 300 °C. Silicon is lightly doped p-type with doping concentration of ∼ 1 × 10^16^. The measurement is performed under illumination to facilitate the polarization switching. **(c)** Illustration of the HCS of the device after programed by the negative voltage at TE. **(d)** Illustration of the LCS of the device after erased by the positive voltage at TE

The first attempt to realize FCM using the MFS structure is shown in Fig. 2 (a) [57]. This device employs the accumulation and depletion of a lightly doped silicon layer to achieve tunable capacitance and is referred to as the accumulation-type FCM to distinguish it from the other type of MFS FCM discussed in the next subsection. The accumulation-type FCM can be fabricated by depositing the ferroelectric layer directly on the silicon surface, followed by a metal capping layer on top as the top electrode (TE). The bottom electrode (BE) is connected to the bulk silicon. Figure 2 (b) plots a C-V curve of an accumulation-type FCM with W top electrode, 10 nm Al-doped HfO_2_ (HAO) ferroelectric film, and lightly doped p-type (p^−^) silicon. Due to the accumulation and depletion of the holes in the silicon layer, the capacitance of the device is high under a negative voltage bias applied at the TE and falls to a low value under a positive bias. The polarization of the ferroelectric layer provides a clockwise hysteresis window to the curve and hence two capacitance states at zero bias. In HCS, attracted by the dipoles after a negative voltage, the holes accumulate to the silicon surface. The device capacitance approaches the capacitance of the ferroelectric layer (*C*_Fe_) as shown in Fig. 2 (c). In LCS illustrated in Fig. 2 (d), the dipoles in the opposite direction repel the holes, leading to a depletion layer in the silicon. Thus, the device capacitance is low due to the depletion capacitance (*C*_Dep_) in series. By using the semiconductor to modulate the capacitance, the accumulation-type FCM is able to offer a capacitance ratio over 100 [61], much higher than that of the MFM FCM.

Although accumulation-type FCM structure significantly enhances the capacitance ratio, experiments indicated that the insufficient minority carriers result in the difficulty in the erase operation (referred to as weak erase issue in this article) [49, 58, 76–78]. When a positive voltage is applied to the TE to erase the FCM to its LCS, a wide depletion region forms in the silicon layer, where a significant portion of the voltage drops. This makes it difficult to induce sufficient electric field in the ferroelectric layer for the polarization switching. For the device demonstrated in Fig. 2 (b), the carrier generation of the p^−^ silicon in the dark environment under room temperature (∼ 298 K) cannot support the erase operation even with 1 s erase pulse width and 5 V amplitude, severely limiting the application of such devices. While, under illumination, the photogenerated carriers in the silicon layer can effectively assist the erase operation of the device. Leveraging the illumination-sensitive operation of the accumulation-type FCM, Ning et al. proposed a ferroelectric optoelectronic memcapacitor [61]. Written by both electrical stimuli and optical stimuli, the interesting device combines the function of optical sensor and memory, demonstrating potential for photoelectric in-memory logic and computing [62].

#### Inversion-type FCM

**Fig. 3:**
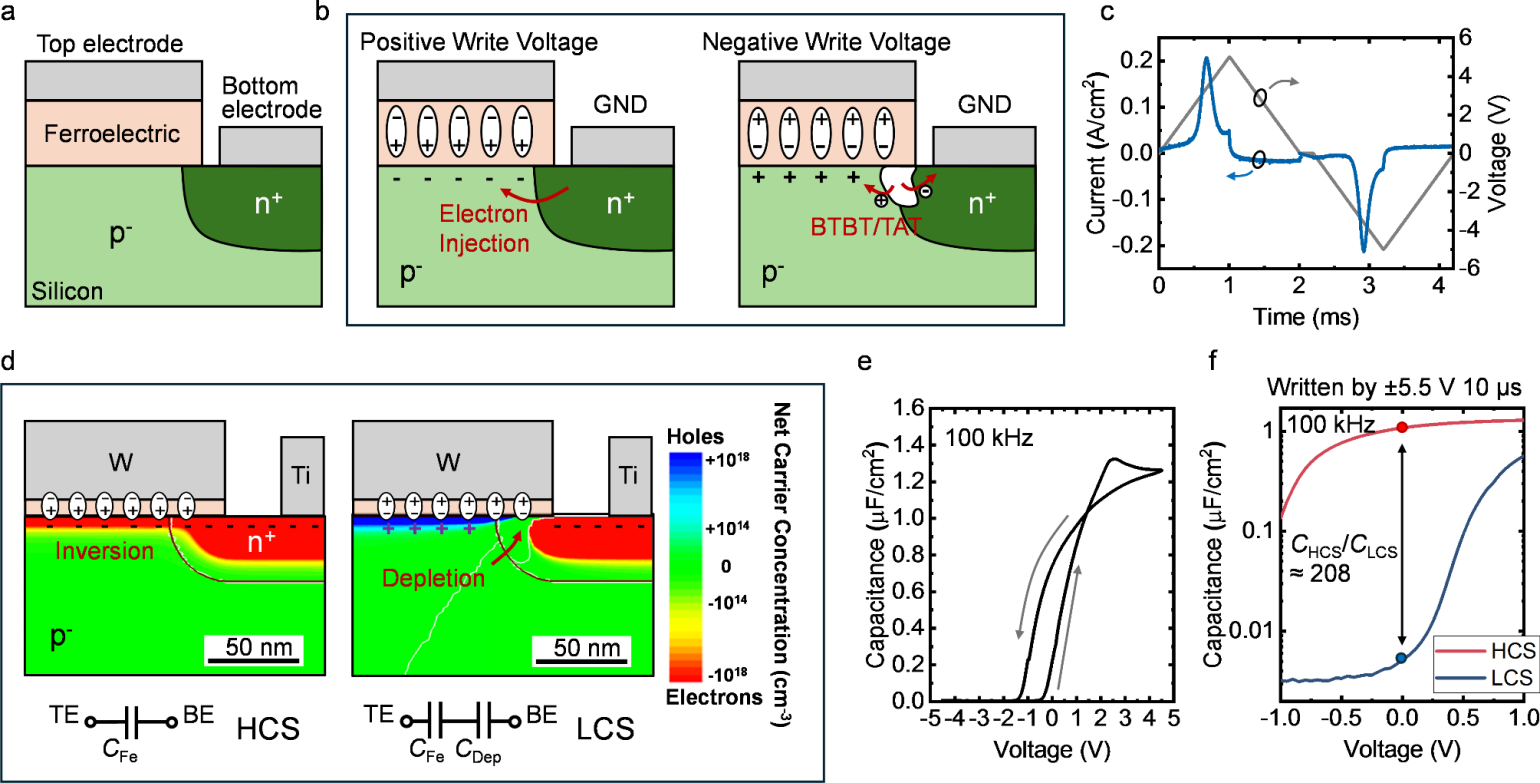
**(a)** Device structure of the inversion-type FCM [58, 59]. **(b)** Illustration of the write operation of inversion-type FCM. **(c)** The transient current response to the triangular voltage pulse, showing effective polarization switching. **(d)** Illustration of the charge distribution in the two memory states of the inversion-type FCM. **(e)** Measured C-V curve of an inversion-type FCM device. The ferroelectric layer is 10 nm HAO deposited by ALD. The n^+^ region is introduced by self-aligned phosphorus implantation. **(f)** Measured C-V curves of the device in two memory states. The device is written by voltage pulses with amplitude of 5.5 V and width of 10 μs, showing a capacitance ratio of ∼ 208 at zero bias

The inversion-type FCM was proposed to address the erase issue of the accumulation-type FCM by introducing a heavily doped region to supply the carriers for polarization switching [58–60]. The device structure is drawn in Fig. 3 (a). Through ion implantation, an n^+^ region is placed under the BE, slightly overlapping with the TE edge of the MFS stack. As illustrated in Fig. 3 (b), this heavily doped region injects minority carriers (electrons) into the silicon surface, assisting the fast polarization switching when written to HCS with a positive voltage at TE. On the other hand, during the writing of LCS with a negative voltage, the strong band bending at the overlap n^+^ region under the TE generates electron-hole pairs by band-to-band tunneling (BTBT) and trap-assisted tunneling (TAT), providing sufficient carriers for the polarization switching in the other direction [49, 59]. As shown in the measurement in Fig. 3 (c), the transient switching current peaks indicate that ferroelectric layer in the device can be effectively switched in both two directions and the weak erase issue is resolved.

The capacitance modulation mechanism in the two memory states utilizes the inversion and depletion of the silicon layer [see Fig. 3 (d)]. Thus, such devices are defined as inversion-type FCM. Specifically, in HCS, the inversion carriers are attracted by the polarization to the silicon surface, hence maintaining the high capacitance. In LCS, the PN junction is in reverse bias. The large depletion region at the junction leads to a low capacitance [59].

The C-V loop plotted in Fig. 3 (e) is measured from an inversion-type FCM with 10 nm HAO ferroelectric layer deposited on p^−^ silicon substrate and an n^+^ region introduced by phosphorus implantation. An anticlockwise hysteresis window can be observed. Figure 3 (f) displays the C-V curves with small sweeping range for the two memory states after write operations with ± 5.5 V amplitude and 10 μs width, showing a large capacitance ratio of ∼ 208 at zero bias. Is should be noted that, typically, the capacitance ratio measured in the device written by short voltage pulses [see Fig. 3 (f)] is larger than that measured in the quasi-DC voltage sweeping case [see Fig. 3 (e)] because of the charge trapping effect at the interface [24, 79–83]. Similar to the FeFET, the interface of the MFS stack plays a critical role in the device performance [50]. Various approaches to improve the interface quality of FeFET gate stack can also be implemented to optimize MFS FCMs [48, 84–89].

Without the erase issue, the write operation of FCM can be fast. Experiments have proved that voltage pulses shorter than 100 ns can effectively change the capacitance state of the device [58]. Additionally, with the dramatically enhanced capacitance ratio and the intrinsic multi-state characteristics of HfO_2_-based ferroelectric materials, the capacitance of the inversion-type FCM is continuously programmable with a large range [58], demonstrating great potential for multi-level memory cell and analog CiM applications.

Compared with MFM FCM, the inversion-type FCM with MFS structure exhibits better memory performance in terms of capacitance ratio and write speed. However, the voltage drop across the semiconductor layer increases the write voltage of the device as listed in Table 1. Strategies including thinning down the ferroelectric layer and optimization of the ferroelectric\semiconductor interface can be applied to achieve lower write voltages for low-power systems. Besides, the application of semiconductors degrades the integration flexibility compared to MFM FCM.

In Addition, the heavily doped region in the device structure introduces an extra factor to consider during the device down scaling. In the inversion-type FCM, the overlapped heavily doped region under TE results in the overlap capacitance (*C*_ov_) from BE to TE. The effect of *C*_ov_ is negligible in the device with large TE. However, since *C*_ov_ is not scaled with TE area, such capacitance can dominate the LCS capacitance of highly scaled inversion-type FCM. This not only degrades the capacitance ratio but also introduces concerns about device variation due to the process-induced diffusion length variation. Therefore, controlling the diffusion length and hence the overlap region is critical in the scaling of inversion-type FCM, which can be done by various approaches including optimization of the annealing process and application of the spacer.

#### Using FeFET as FCM

**Fig. 4:**
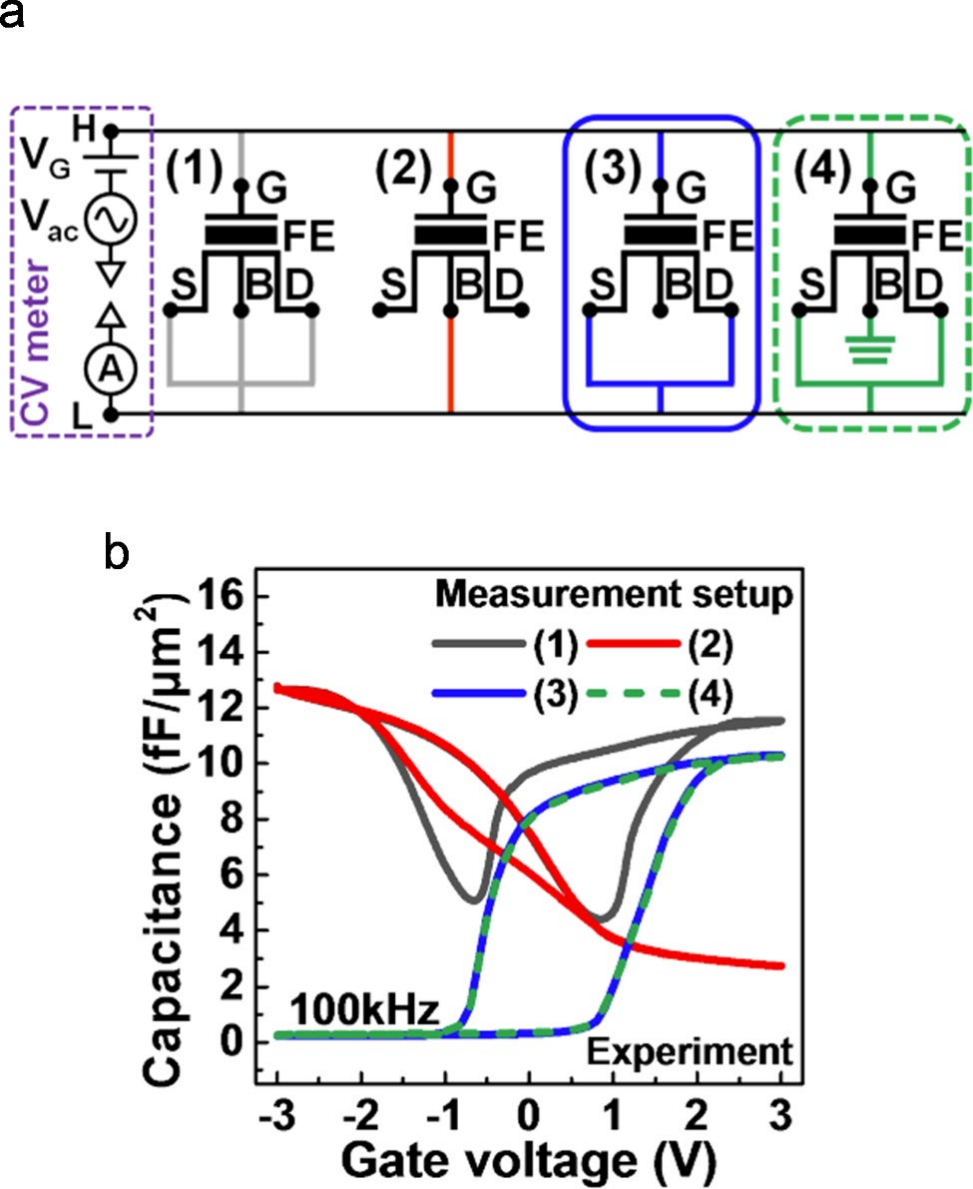
**(a)** Four different setups for the measurement of the FeFET capacitance. **(b)** Measured C-V curves with different setups. Reproduced with permission [63]

By leveraging the MFS gate stack, FeFET can also be accessed as the capacitive memory. Kim et al. and Phadke et al. have demonstrated the capacitance modulation capability of the n-type Si FeFETs on the GlobalFoundaries’ 28 nm platform [42, 63, 64]. Four measurement setups shown in Fig. 4 (a) were investigated to obtain the C-V hysteresis curves of FeFETs. With different terminals connected, carrier response to the high-frequency small signal can vary, leading to the four C-V curves displayed in Fig. 4 (b) [63]. In setup (1), both electrons and holes can respond to the small signal. Thus, the capacitance is high for both negative and positive voltage biases. In this setup, although the hysteresis window of the C-V curve is large, the capacitance ratio is relatively low. Setup (2) measures the gate-to-body capacitance of FeFETs. Without connecting the source and drain regions, this setup is equivalent to an accumulation-type FCM, where the weak erase issue occurs. By connecting the heavily doped source/drain region and leaving the body terminal floating, setup (3) configures the FeFET as an inversion-type FCM. With the same mechanism discussed before, the measured C-V curve presents a large capacitance ratio at zero bias. Similar results can also be obtained with setup (4), where the body terminal is grounded. With this setup, the holes from the silicon bulk can be expected to assist the polarization switching and may accelerate erase operation. The demonstration of FCM operation in the FeFETs indicates a seamless transition from the intensively researched FeFET technologies to FCM. Various fabrication processes and device optimization strategies developed for FeFETs can be migrated to FCMs. Besides, the capability of both resistive and capacitive reading makes FeFET a versatile device with enhanced application compatibility.

**Table 1:** Various FCM devices in the existing reports. Capacitance ratio is read at zero bias if not specified. Retention is estimated by extrapolation

Device Type	Reference	Device Structure	Write Voltage	Write Speed	Capacitance Ratio	Retention	Endurance	Remarks
**MFM**	Zheng and Wang et al. [51]	TiN/HAO/TiN	± 4 V	-	∼ 1.01 (@ ∼-1.6 V)	-	-	Symmetric C-V, no ratio @ 0 V bias
	Luo et al. [52]	TiN/HZO/TiN	± 3 V	1 ms	∼ 1.13	10 years under 85 °C	∼ 1E4	-
	Luo and Hur et al. [53]	TiN/HZO/TiN	± 3 V	1 ms	∼ 1.15	10 years under 85 °C	∼ 1E3	12 × 12 crossbar array
	Mukherjee et al. [54]	Mo + MoO_*x*_/La: HZO/TiN	± 3 V	-	∼ 1.16	10 years under 85 °C	-	-
	Xu and Fu et al. [55]	TiN/HZO/TiN	± 3 V	-	∼ 1.13 (@ ∼0.8 V)	10 years	∼ 1E9	Symmetric C-V, no ratio @ 0 V bias
	Yu and K et al. [56]	TiN/HZO/ZrO_2_/TiN	± 1.2 V	-	∼ 1.22 (@ ∼0.6 V)	-	-	4.5 nm HZO, small ratio @ 0 V bias
		TiN/HZO/HfO_2_/TiN	± 4 V	-	∼ 1.30 (@ ∼1 V)	-	-	9.5 nm HZO, small ratio @ 0 V bias
	This work	W/HZO/W	± 3 V	-	∼ 1.15	-	-	-
**MFS** **(Accu.-type)**	Zhou and Zhou et al. [57]	W/HAO/p^−^ Si	+ 3 V, -5 V	1 ms, 1 μs	∼ 5	10 years	∼ 1E3	Measured under illumination due to weak erase issue
	Liu et al. [61] Liu et al. [62]	WN/HAO/SiON/p^−^ Si	+ 5 V, -6 V	1 ms, 1 μs	∼ 134	10 years	∼ 1E8	Optoelectronic memory, written by both electrical and optical stimuli
**MFS** **(Inversion-type)**	Kim, Phadke, and Luo et al. [63] Phadke et al. [64]	TiN/Si: HfO_2_/SiON/p^−^ Si	+ 3.5 V, -4.5 V	700 ns	∼ 25	10 years under 85 °C	∼ 1E8 (by recovery)	Gate to source/drain capacitance of GlobalFoundries’ 28 nm FeFET [42]
	Zhou and Jiao [58] Zhou and Jiao [59]	W/HAO/p^−^ Si	± 5 V to ± 6.5 V	50 ns to 1 μs	∼ 125 (@ ∼-0.2 V) ∼ 80 (@ 0 V)	10 years under 85 °C	∼ 1E5	32 × 32 crossbar array
	Zhou and Jiao [60]	W/HAO/p^−^ Si	± 5.5 V	10 μs	∼ 11 (@ ∼-0.3 V) ∼ 7 (@ 0 V)	10 years	∼ 5E5	Monolithic 3D stacked 1T1C memory cell
	This work	W/HAO/p^−^ Si	± 5.5 V	10 μs	∼ 208	-	-	-

## Array-level operation & integration demonstration

With the unique capacitive reading scheme, the FCM operation at the array level can be fundamentally different from that of conventional resistive memories. In this section, we compare the two types of crossbar arrays, showing the mitigated sneak path and IR drop issues in the capacitive array with specific connection schemes. Various experimental demonstrations of FCM integration and array-level operation are also presented.

### Capacitive crossbar array

In this article, the floating bit line (BL) and grounded BL connection schemes of the crossbar arrays are defined by the connection of the unselected BLs. In these two schemes compared and discussed in this subsection, the unselected word lines (WLs) are floating. The connection schemes with the unselected WLs grounded can be analyzed in the same manner, which exhibits the effects similar to those of the grounded BL connection scheme.

**Fig. 5:**
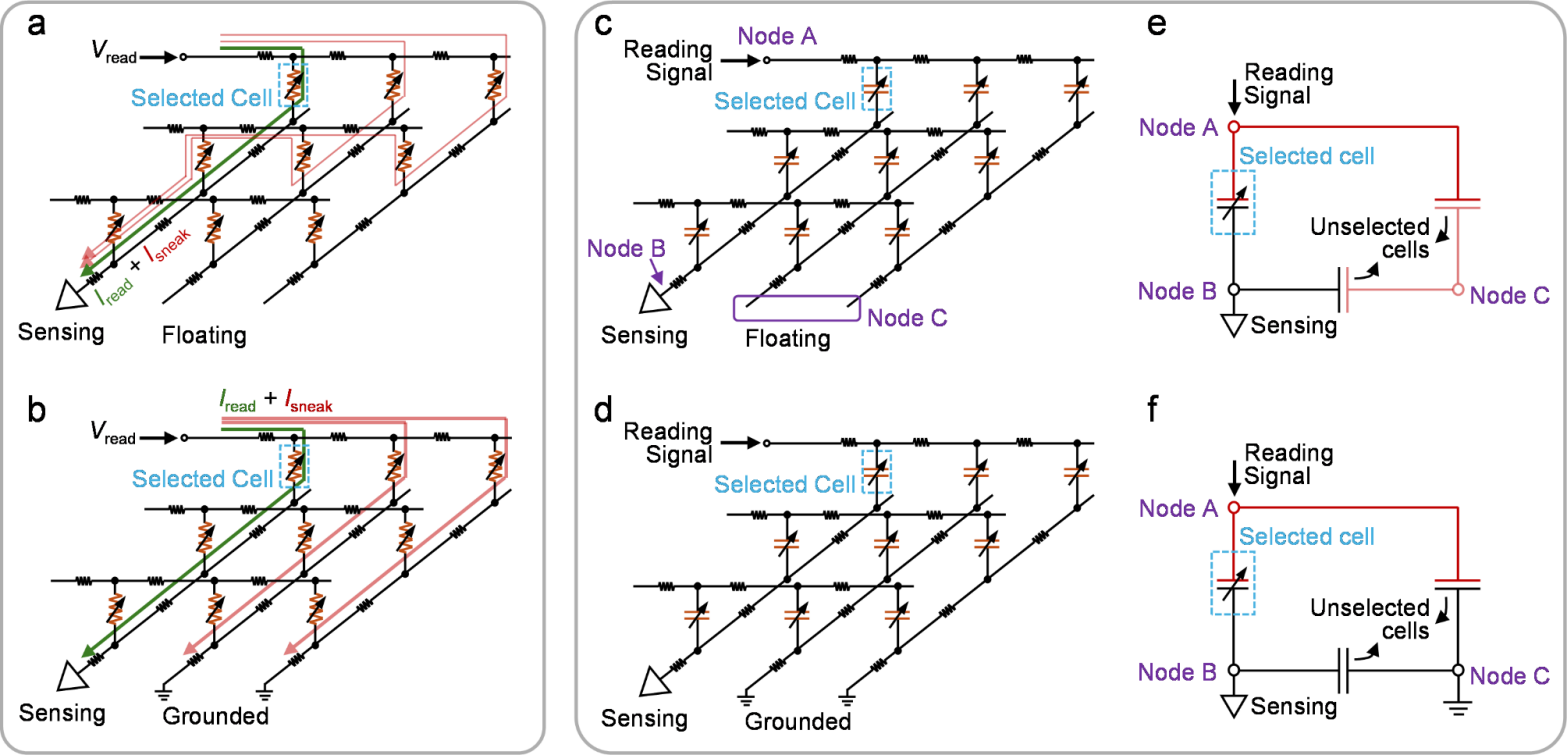
**(a)** Illustration of the sneak path issue in the resistive crossbar array with floating BL connection scheme. **(b)** Illustration of the IR drop issue in the resistive crossbar array with grounded BL connection scheme. **(c)** Floating BL connection scheme and **(d)** grounded BL connection scheme of the capacitive crossbar array. **(e)** and **(f)** simplified equivalent circuits of the capacitive crossbar array with floating BL connection scheme and grounded BL connection scheme, respectively. The effect of the unselected cells in the capacitor network is simplified as two capacitors. One is connected between Node A (selected WL) and Node C (unselected BLs). The other one is connected between Node B (selected BL) and Node C. Interconnect resistance is ignored in the simplified equivalent circuits

**Fig. 6:**
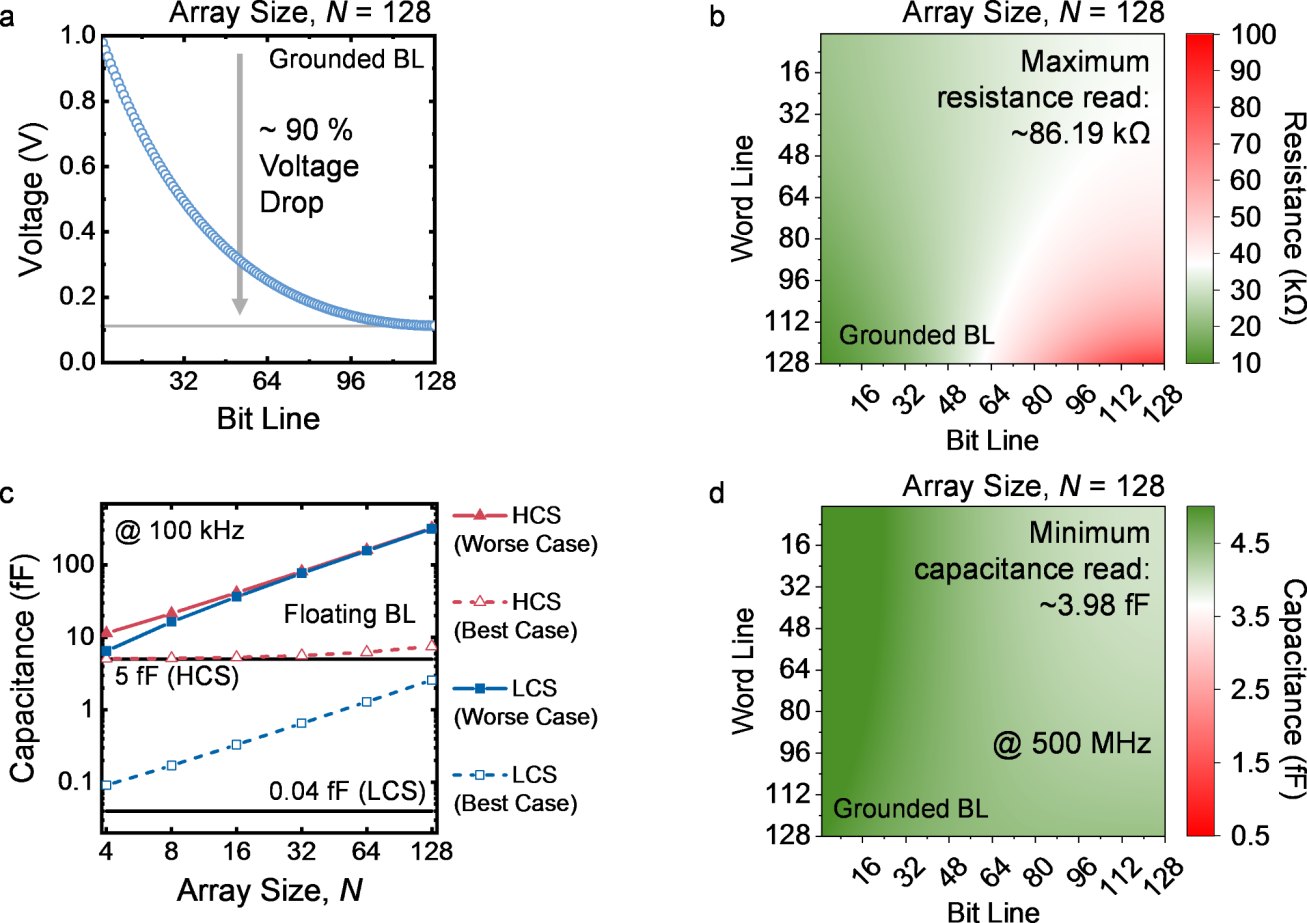
**(a)** Voltages at the top electrodes of the resistive memory devices connected to the 128th WL in a 128 × 128 resistive crossbar array with grounded BL connection scheme. All the devices are in low resistance state. **(b)** Resistance read from a 128 × 128 resistive crossbar array with grounded BL connection scheme and all the devices in low resistance state. **(c)** Reading of one memory device in the *N* × *N* capacitive crossbar array with different array size, *N*. The BLs are floating. The best case is defined as a scenario where all the unselected cells are in the LCS. The worst case is defined as a scenario where all the unselected cells are in the HCS. **(d)** Capacitance read from a 128 × 128 capacitive crossbar array with grounded BL connection scheme under 500 MHz. All the devices are in HCS. In the above simulations, interconnect resistance between two adjacent devices is set to 5 Ω. Resistance of the resistive memory device is assumed to be 10 kΩ for low resistance state. Capacitive memory devices are assumed to have a capacitance of 5 fF and 0.04 fF for HCS and LCS, respectively

Figure 5 (a) shows the read operation to access a selected cell in the resistive crossbar array with the floating BL connection scheme. In this case, in addition to the current flowing through the selected memory cell, the read voltage (*V*_read_) at the selected WL also induces sneak current flowing into the sensing circuit via multiple sneak paths, resulting in the reading error. The sneak path issue significantly narrows the read margin and becomes more pronounced as the array size is scaled up. While, with the unselected BLs grounded, the current in the array will dramatically increase, which not only degrades the energy efficiency but also leads to severe IR drop issue. In the simulation of a 128 × 128 resistive crossbar array with grounded BL connection scheme, ∼ 90% of the applied voltage drops across the parasitic resistance along the interconnect [see Fig. 6 (a)]. The insufficient voltage delivered to the memory device causes large reading errors especially for the devices far from voltage source. As shown in Fig. 6 (b), the reading of the cell resistance can be several times higher than the expected value in the worst-case scenario. Therefore, selectors are often required to form the 1-selector-1-resistor memory cells for the resistive crossbar arrays [71, 72, 90–92].

For the capacitive crossbar array, a similar sneak path issue occurs when the unselected BLs are floating. As illustrated in the schematic in Fig. 5 (c) and a simplified equivalent circuit in Fig. 5 (e), the unselected memory cells connected in parallel with the selected cell in the floating BL connection scheme, leading to a higher capacitance sensed between node A and B. Figure 6 (c) displays the simulation results of reading a capacitive memory cell in the crossbar arrays with different array sizes, showing that the sneak path issue closes the sensing margin even for the small arrays [59]. In comparison, by fully utilizing the properties of the memory device, the grounded BL connection scheme drawn in Fig. 5 (d) enables accurate reading of capacitive crossbar array without the incorporation of selectors. As shown in the simplified equivalent circuit in Fig. 5 (f), the grounded BLs disconnect the signal paths in parallel with the selected cell by deactivating the unselected capacitors between node B and C, which circumvents the sneak path issue. Different from the situation in resistive crossbar arrays, the grounded BL connection scheme of the capacitor network does not introduce direct current path to ground, which prevents the surge in direct current and hence maintains the low-power reading operation. Furthermore, thanks to the ultra-high resistance of the capacitive memory devices, the IR drop issue is significantly alleviated. Therefore, the voltage signal can be effectively applied on the devices for the accurate capacitance sensing. However, it should be noted that the interconnect resistance still limits the bandwidth of the array, which can lead to reading errors at high frequencies [59]. As estimated in the simulation shown in Fig. 6 (d), under a 500 MHz reading signal, reading errors can be observed in the worst-case scenario with 5 Ω interconnect resistance. Thus, to ensure the high-frequency reading operation, control of parasitic resistance in the capacitive memory crossbar array is necessary.

**Fig. 7:**
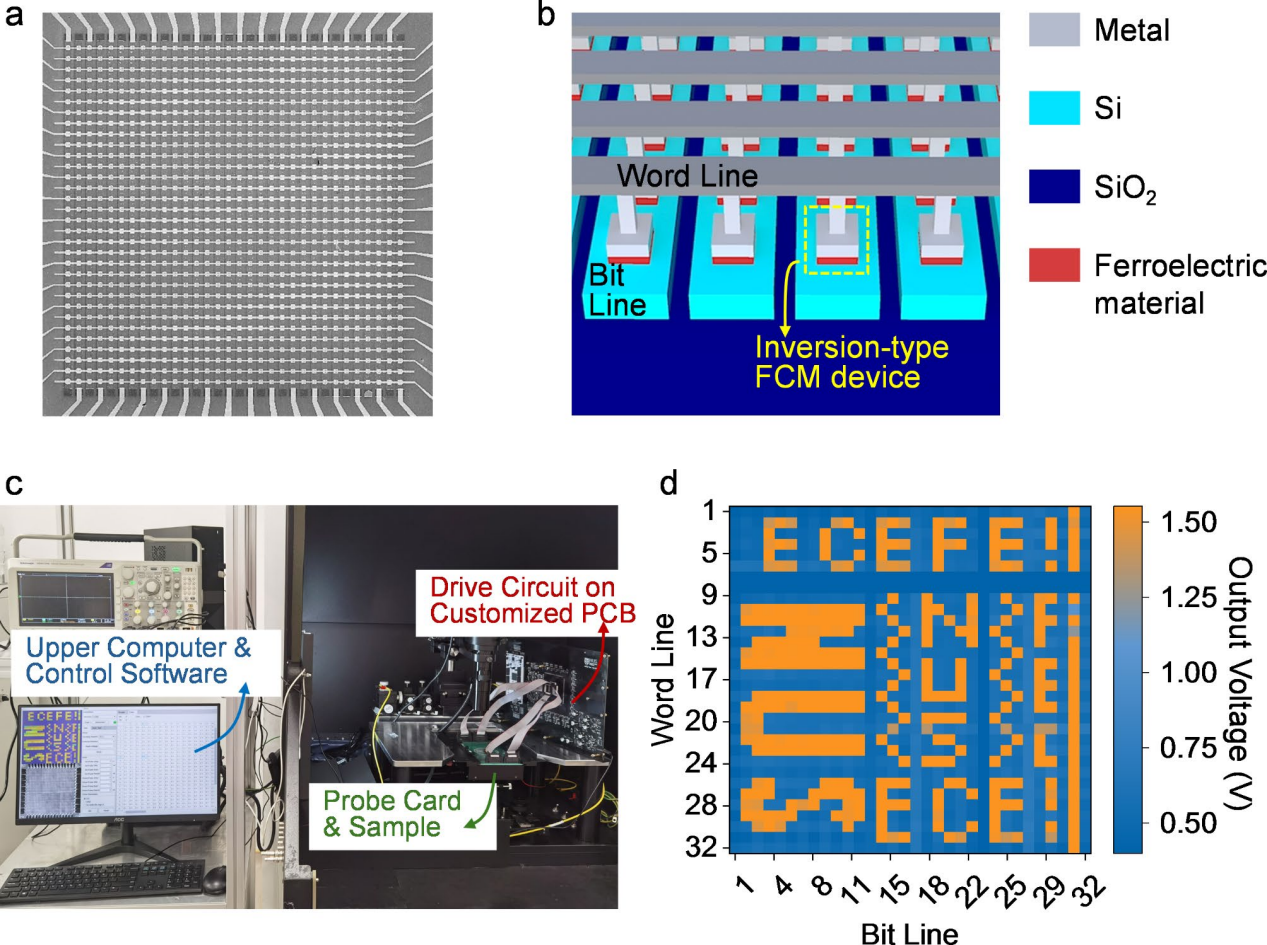
**(a)** Scanning electron microscope image of the fabricated capacitive crossbar array based on inversion-type FCM [58, 59]. **(b)** Structure of the array. **(c)** Measurement setup for the capacitive crossbar array. **(d)** Reading output of the array after programmed with an image pattern

The integration of capacitive memory devices into crossbar arrays has been experimentally demonstrated based on different FCM designs. Luo and Hur et al. reported a 12 × 12 crossbar array based on MFM FCM devices with TiN/HZO/TiN stack [53]. A 1/3 write voltage (*V*_w_) write scheme was applied to suppress the disturbance among the memory cells during the array-level write operation. Additionally, an external charge-transfer circuit based on the operational amplifiers was designed for array sensing with short voltage pulse input. In the experiments, the array-level multiply-accumulate (MAC) operation was presented. Demasius et al. fabricated a 26 × 6 crossbar array with the capacitive memory devices based on charge screening mechanism [93]. A similar 1/3 *V*_w_ scheme was implemented for the write operation. The array-level read operation was realized by using AC voltage input signals. The crossbar array integration of inversion-type FCM was demonstrated by Zhou and Jiao et al. on the silicon-on-insulator (SOI) platform [58]. Figure 7 (a) displays the scanning electron microscope image of a fabricated 32 × 32 array. As depicted in Fig. 7 (b), to integrate the FCM with MFS stack, the silicon layer was etched to isolate the devices in different columns, and the heavily doped regions of the silicon bars introduced via ion implantation connected the devices in each row, forming the BLs. Next, metal lines on top of a thick isolation layer connected the TEs of the devices as the WLs. The fabricated arrays were measured by a specific test platform shown in Fig. 7 (c), where 1/3 *V*_w_ write scheme was implemented to program the array and a charge-transfer circuit was built for the array-level sensing. With the grounded BL connection scheme, the output voltages of the sensing circuit for the reading of each memory cell in the array are drawn in Fig. 7 (d). The pre-written image pattern in the array can be clearly seen, proving the successful demonstration of the array-level write and read operations [58].

It is noteworthy that, different from the resistive memory, FCMs are more susceptible to the effect of parasitics capacitance due to their capacitive reading mechanisms. Although FCMs exhibit immunity to the IR drop issue, the reading may be limited by the parasitic capacitance in the array or circuit. As the studies of FCMs are still in the relatively early stage, most of existing studies are on large devices, where the effect of parasitics capacitance is negligible. Because the FCM capacitance is scaled with the device area, optimization of the parasitics capacitance is important for the reading of highly scaled FCMs. On the other hand, to mitigate the effect of parasitics, several approaches can be implemented to increase the FCM capacitance. For example, the ferroelectric layer with thinner thickness can be applied to increase the capacitance density of the device. The 3D trench or pillar capacitor structures similar to the advanced DRAM or FeRAM technologies can also be adapted to FCMs to provide a larger effective capacitor area within the same footprint. Further studies are necessary to explore the scalability of FCMs at both device level and array level.

### Integration with access transistors

Although capacitive memory crossbar arrays are able to avoid the sneak path and IR drop issues with the grounded BL connection scheme, charging a large number of devices in parallel increases the latency and energy consumption when the array size is scaled up [59, 60]. Besides, similar to the resistive memories, the unselected FCM devices inside the arrays suffer from write disturbance even under the partial bias schemes [53]. To address these issues and pave the way for the large-scale FCM arrays, the access transistors can be integrated with the FCM devices to form the 1T1C memory cells.

**Fig. 8:**
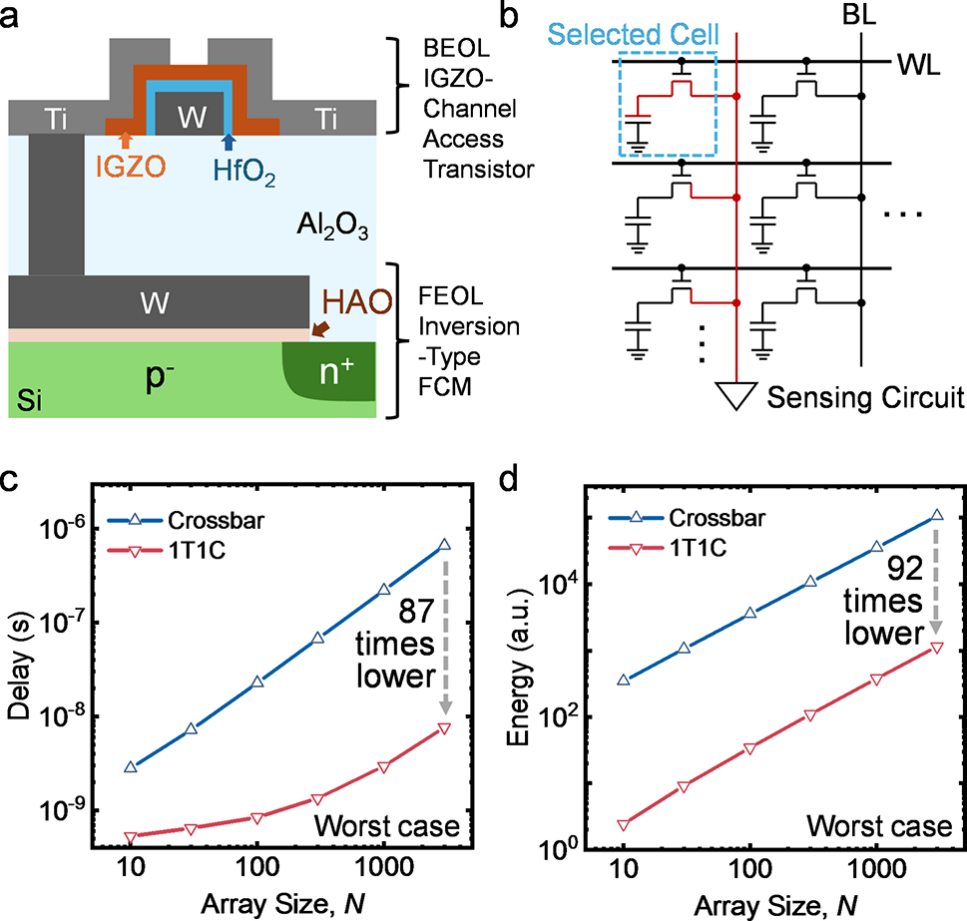
**(a)** Cross-section structure of the 1T1C FCM memory cell [60]. **(b)** Schematic of 1T1C FCM memory array. **(c)** Comparison of the delay of crossbar array and 1T1C array. The delay is defined as the time to reach 90% of WL or BL voltage. **(d)** Comparison of the energy consumption of crossbar array and 1T1C array

By vertically stacking the inversion-type FCM with the indium gallium zinc oxide (IGZO) channel access transistor on BEOL, Zhou and Jiao et al. fabricated a 1T1C FCM cell and demonstrated the cell operation [60]. By leveraging the monolithic 3D integration capacity of IGZO transistor, the access transistor shares the same footprint with the memory device, as shown in Fig. 8 (a). In the memory array [see Fig. 8 (b)], the access transistors effectively isolate the unselected cell from the active BL. As compared in Fig. 8 (c) and (d), the introduction of access transistors not only eliminates the write disturbance but also dramatically lowers the delay and energy consumption of the read operation especially for the large-scale arrays [60].

## Capacitive synapses for CiM applications

**Fig. 9:**
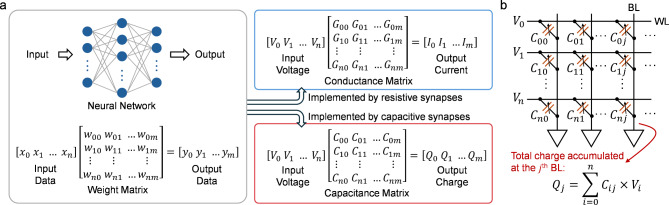
**(a)** Comparison of MAC operations for neural network acceleration implemented by resistive synapses and capacitive synapses. **(b)** Charge-domain MAC operation based on a *n* × *m* FCM crossbar array

As formulated in Fig. 9 (a), the MAC operations play a critical role in the neural-network-based machine learning, which can be effectively accelerated using CiM paradigm. By utilizing resistive memories as synapse devices, the weights (*w*_*ij*_) in the network are mapped to the conductance (*G*_*ij*_) of the devices, and the resistive crossbar array outputs the MAC results as the currents. This computation can be transferred to charge domain by using FCMs as capacitive synapses. With device capacitance (*C*_*ij*_) representing the weights, the amount of charge at each device induced by the applied voltages is equal to the product of corresponding input and weight. Therefore, as shown in Fig. 9 (b), the total charge accumulated at each BL can be sensed to determine the MAC results.

In addition to the eliminated data movement and high parallelism enabled by the CiM architecture, the properties of capacitive memory crossbar array further lower the energy consumption and improve the area efficiency of the accelerator. Zheng and Wang et al. proposed the application of the varying capacitance of symmetric MFM capacitors in the neural network acceleration in 2019 [51]. In 2021, Luo and Hur et al. experimentally implemented the MAC operations in the 12 × 12 crossbar array based on MFM FCM with capacitance ratio at zero DC bias [53]. In the experiments, a charge transfer circuit was applied to sense the total amount of charge at BLs with input voltage pulses. The simulation predicted a 20–200 times improvement in the energy consumption compared with the resistive memory devices at the subarray-level. The system-level evaluation further presented the outstanding performance of the capacitive crossbar array compared with static random-access memory (SRAM) at advanced technology nodes, including smaller chip area, shorter latency, and lower power consumption. Besides, Demasius et al. experimentally demonstrated a 5 × 5 image recognition task implemented in the capacitive crossbar array with high capacitance ratio [93]. To realize stimulus and inhibition inputs as well as positive and negative weights, a ‘four-quadrant multiplication’ strategy specifically designed for capacitive crossbar array was proposed by encoding the input as AC voltage signals with 0°/180° phase shifts and sensing the positive/negative weight cells in two phases. Simulations indicated that the capacitive crossbar arrays have the potential to realize 1,000–10,000 TOPS/W energy efficiency, 10–100 times higher than the human brain or other resistive memories.

## Conclusion

With the capacitive reading mechanism, FCMs exhibit various remarkable superiorities over the conventional resistive memories, including better energy efficiency, immunity to IR drop or sneak path issue, and low read disturbance, which also demonstrate great potential as the fundamental building blocks for charge-domain CiM systems. In this article, several typical FCM designs are introduced from the device-level capacitance modulation mechanisms to the array-level integration and demonstrations. In addition, the CiM applications of the capacitive crossbar arrays are presented, showing the outstanding performance at system level. However, as technology developed in recent years, FCMs are relatively immature compared with their resistive counterparts. The performance of current devices can be further optimized by various approaches to realize better capacitance ratio, reliability, and lower operation voltages. Besides, the FCM devices with nanoscale sizes require further exploration with both experiments and theoretical investigations to better prove the scalability of such memories. Integration strategies can be developed to control the effect of parasitics and enable higher integration density. Moreover, the charge-domain operations also necessitate further studies in the peripheral circuit design to realize more efficient capacitive memory and CiM operations. Toward the next-generation memories for future data storage and computing applications, co-optimization at different levels is essential to fully unleash the capabilities of the FCM technologies.

## Data Availability

The review is based on the published data and sources of data upon which conclusions have been drawn can be found in the reference list.

## References

[1] S. Jung et al., A crossbar array of magnetoresistive memory devices for in-memory computing. Nature. **601**, 211–216 (2022). 10.1038/s41586-021-04196-6 35022590 10.1038/s41586-021-04196-6

[2] P. Yao et al., Face classification using electronic synapses. Nat. Commun. **8**, 1–8 (2017). 10.1038/ncomms15199 28497781 10.1038/ncomms15199 PMC5437298

[3] P. Yao et al., Fully hardware-implemented memristor convolutional neural network. Nature. **577**, 641–646 (2020). 10.1038/s41586-020-1942-4 31996818 10.1038/s41586-020-1942-4

[4] B. Gao et al., Memristor-based analogue computing for brain-inspired sound localization with in situ training. Nat. Commun. **13** (2022). 10.1038/s41467-022-29712-8 10.1038/s41467-022-29712-8 PMC9018844 35440127

[5] W. Zhang et al., Neuro-inspired computing chips. Nat. Electron. **3**, 371–382 (2020). 10.1038/s41928-020-0435-7

[6] J.M. Hung, X. Li, J. Wu, M.F. Chang, Challenges and trends in developing nonvolatile memory-enabled computing chips for intelligent edge devices. IEEE Trans. Electron. Devices. **67**, 1444–1453 (2020). 10.1109/TED.2020.2976115

[7] K. Ni et al., Ferroelectric ternary content-addressable memory for one-shot learning. Nat. Electron. **2**, 521–529 (2019). 10.1038/s41928-019-0321-3

[8] S. Yu, Neuro-inspired computing with emerging nonvolatile memory. *Proc. IEEE* 106, 260–285 (2018). 10.1109/JPROC.2018.2790840

[9] P. Jain et al., 13.2 A 3.6 Mb 10.1 Mb/mm^2^ embedded non-volatile ReRAM macro in 22nm FinFET technology with adaptive forming/set/reset schemes yielding down to 0.5V with sensing time of 5ns at 0.7V. in *2019 IEEE International Solid-State Circuits Conference (ISSCC)*. 2019. IEEE. 10.1109/ISSCC.2019.8662393

[10] J.Y. Wu et al., A 40nm low-power logic compatible phase change memory technology. in. 2018 *IEEE International Electron Devices Meeting (IEDM).* 2018. IEEE. 10.1109/IEDM.2018.8614513

[11] V.B. Naik et al., Manufacturable 22nm FD-SOI embedded MRAM technology for industrial-grade MCU and IOT applications. in *2019 IEEE International Electron Devices Meeting (IEDM).* 2019. IEEE. 10.1109/IEDM19573.2019.8993454

[12] N. Raghavan et al., Stochastic variability of vacancy filament configuration in ultra-thin dielectric RRAM and its impact on OFF-state reliability. in. 2013 *IEEE International Electron Devices Meeting (IEDM).* 2013. IEEE. 10.1109/IEDM.2013.6724674

[13] S. Yu, X. Guan, H.S.P. Wong, On the stochastic nature of resistive switching in metal oxide RRAM: Physical modeling, Monte Carlo simulation, and experimental characterization. in 2011 *IEEE International Electron Devices Meeting (IEDM).* 2011. IEEE. 10.1109/IEDM.2011.6131572

[14] S.H. Lee et al., Programming disturbance and cell scaling in phase change memory: For up to 16nm based 4F^2^ cell. in *2010 IEEE Symposium on VLSI Technology (VLSI)*. 2010. IEEE. 10.1109/VLSIT.2010.5556226

[15] S.J. Ahn et al., Reliability perspectives for high density PRAM manufacturing. in. 2011 *IEEE International Electron Devices Meeting (IEDM).* 2011. IEEE. 10.1109/IEDM.2011.6131542

[16] T. Kim, S. Lee, Evolution of phase-change memory for the storage-class memory and beyond. IEEE Trans. Electron. Devices. **67**, 1394–1406 (2020). 10.1109/TED.2020.2964640

[17] S. Slesazeck, T. Mikolajick, Nanoscale resistive switching memory devices: A review. Nanotechnology. **30**, 352003 (2019). 10.1088/1361-6528/ab2084 31071689 10.1088/1361-6528/ab2084

[18] S. Yu, P.Y. Chen, Emerging memory technologies: Recent trends and prospects. IEEE Solid-State Circuits Mag. **8**, 43–56 (2016). 10.1109/MSSC.2016.2546199

[19] T.S. Böscke, J. Müller, D. Bräuhaus, U. Schröder, U. Böttger, Ferroelectricity in hafnium oxide thin films. Appl. Phys. Lett. **99** (2011). 10.1063/1.3634052

[20] T.S. Böescke, J. Müller, D. Bräuhaus, U. Schröder, U. Böttger, Ferroelectricity in hafnium oxide: CMOS compatible ferroelectric field effect transistors. in. 2011 *IEEE International Electron Devices Meeting (IEDM).* 2011. IEEE. 10.1109/IEDM.2011.6131606

[21] A.I. Khan, A. Keshavarzi, S. Datta, The future of ferroelectric field-effect transistor technology. Nat. Electron. **3**, 588–597 (2020). 10.1038/s41928-020-00492-7

[22] F. Huang et al., First observation of ultra-high polarization (108 μC/cm^2^) in nanometer scaled high performance ferroelectric HZO capacitors with Mo electrodes. In *2023 IEEE Symposium on VLSI Technology (VLSI)*. 2023. IEEE. 10.23919/VLSITechnologyandCir57934.2023.10185240

[23] H.J. Lee et al., Scale-free ferroelectricity induced by flat phonon bands in HfO_2_. Science. **369**, 1343–1347 (2020). 10.1126/science.aba0067 32616670 10.1126/science.aba0067

[24] Z. Zhou et al., Investigation of charge trapping aggravation induced by antiferroelectric switching with a unified ferroelectric and antiferroelectric model. IEEE Trans. Electron. Devices. **71**, 4445–4452 (2024). 10.1109/TED.2024.3403084

[25] S.S. Cheema et al., Enhanced ferroelectricity in ultrathin films grown directly on silicon. Nature. **580**, 478–482 (2020). 10.1038/s41586-020-2208-x 32322080 10.1038/s41586-020-2208-x

[26] D. Zhang et al., Grain size reduction of ferroelectric HZO enabled by solid phase epitaxy (SPE): Working principle, experimental demonstration, and theoretical understanding. IEEE Trans. Electron. Devices. **70**, 6665–6672 (2023). 10.1109/TED.2023.3317007

[27] H. Mulaosmanovic et al., Evidence of single domain switching in hafnium oxide based FeFETs: Enabler for multi-level FeFET memory cells. in. 2015 *IEEE International Electron Devices Meeting (IEDM).* 2015. IEEE. 10.1109/IEDM.2015.7409777

[28] M.H. Park et al., Ferroelectricity and antiferroelectricity of doped thin HfO_2_-based films. Adv. Mater. **27**, 1811–1831 (2015). 10.1002/adma.201404531 25677113 10.1002/adma.201404531

[29] J. Zhou et al., Demonstration of ferroelectricity in Al-doped HfO_2_ with a low thermal budget of 500°C. IEEE Electron. Device Lett. **41**, 1130–1133 (2020). 10.1109/LED.2020.2998355

[30] U. Schroeder, M.H. Park, T. Mikolajick, C.S. Hwang, The fundamentals and applications of ferroelectric HfO_2_. Nat. Rev. Mater. **7**, 653–669 (2022). 10.1038/s41578-022-00431-2

[31] N. Ramaswamy et al., NVDRAM: A 32Gb dual layer 3D stacked non-volatile ferroelectric memory with near-DRAM performance for demanding AI workloads. in *2023 IEEE International Electron Devices Meeting (IEDM).* 2023. IEEE. 10.1109/IEDM45741.2023.10413848

[32] T. Francois et al., 16 kbit HfO_2_:Si-based 1T-1 C FeRAM arrays demonstrating high performance operation and solder reflow compatibility. in. 2021 *IEEE International Electron Devices Meeting (IEDM).* 2021. IEEE. 10.1109/IEDM19574.2021.9720640

[33] J. Okuno et al., SoC compatible 1T1C FeRAM memory array based on ferroelectric Hf_0.5_Zr_0.5_O_2_. in *2020 IEEE Symposium on VLSI Technology (VLSI)*. 2020. IEEE. 10.1109/VLSITechnology18217.2020.9265063

[34] P.J. Liao et al., Characterization of fatigue and its recovery behavior in ferroelectric HfZrO. in 2021 IEEE Symposium on VLSI Technology (VLSI). 2021. IEEE

[35] T. Gong et al., 10^5^× endurance improvement of FE-HZO by an innovative rejuvenation method for 1z node NV-DRAM applications. in *2021 IEEE Symposium on VLSI Technology (VLSI)*. 2021. IEEE

[36] F. Mo, Y. Tagawa, T. Saraya, T. Hiramoto, M. Kobayashi, Scalability study on ferroelectric-HfO_2_ tunnel junction memory based on non-equilibrium Green function method with self-consistent potential. in 2018 *IEEE International Electron Devices Meeting (IEDM).* 2018. IEEE. 10.1109/IEDM.2018.8614702

[37] M. Si et al., A novel scalable energy-efficient synaptic device: Crossbar ferroelectric semiconductor junction. in. 2019 *IEEE International Electron Devices Meeting (IEDM).* 2019. IEEE. 10.1109/IEDM19573.2019.8993622

[38] L. Jiao et al., BEOL-compatible Ta/HZO/W ferroelectric tunnel junction with low operating voltage targeting for low power application. in Proceedings of 2022 IEEE International Conference on IC Design and Technology (ICICDT). 2022. IEEE. 10.1109/ICICDT56182.2022.9933091

[39] Z. Zhou et al., Time-dependent Landau-Ginzburg equation-based ferroelectric tunnel junction modeling with dynamic response and multi-domain characteristics. IEEE Electron. Device Lett. **43**, 158–161 (2022). 10.1109/LED.2021.3128998

[40] R. Berdan et al., Low-power linear computation using nonlinear ferroelectric tunnel junction memristors. Nat. Electron. **3**, 259–266 (2020). 10.1038/s41928-020-0405-0

[41] M. Jerry et al., Ferroelectric FET analog synapse for acceleration of deep neural network training. in. 2018 *IEEE International Electron Devices Meeting (IEDM).* 2018. IEEE. 10.1109/IEDM.2017.8268338

[42] M. Trentzsch et al., A 28nm HKMG super low power embedded NVM technology based on ferroelectric FETs. in. 2017 *IEEE International Electron Devices Meeting (IEDM).* 2017. IEEE. 10.1109/IEDM.2016.7838397

[43] S. Dünkel et al., A FeFET based super-low-power ultra-fast embedded NVM technology for 22nm FDSOI and beyond. in. 2018 *IEEE International Electron Devices Meeting (IEDM).* 2018. IEEE. 10.1109/IEDM.2017.8268425

[44] Z. Zhou et al., First study of the charge trapping aggravation induced by anti-ferroelectric switching in the MFIS stack. in 2023 IEEE Symposium on VLSI Technology (VLSI). 2023. IEEE. 10.23919/VLSITechnologyandCir57934.2023.10185236

[45] Y. Feng et al., First demonstration of BEOL-compatible 3D vertical FeNOR. in 2024 IEEE Symposium on VLSI Technology (VLSI). 2024. IEEE. 10.1109/VLSITechnologyandCir46783.2024.10631352

[46] Z. Zheng et al., BEOL-compatible MFMIS ferroelectric/anti-ferroelectric FETs - Part I: Experimental results with boosted memory window. IEEE Trans. Electron. Devices. **71**, 1827–1833 (2024). 10.1109/TED.2023.3326116

[47] Q. Kong et al., Back-end-of-line-compatible fin-gate ZnO ferroelectric field-effect transistors. IEEE Trans. Electron. Devices. **70**, 2059–2066 (2023). 10.1109/TED.2023.3242852

[48] A.J. Tan et al., Ferroelectric HfO_2_ memory transistors with high-κ interfacial layer and write endurance exceeding 10^10^ cycles. IEEE Electron. Device Lett. **42**, 994–997 (2021). 10.1109/LED.2021.3083219

[49] F. Mo et al., Critical role of GIDL current for erase operation in 3D vertical FeFET and compact long-term FeFET retention model. in 2021 IEEE Symposium on VLSI Technology (VLSI). 2021. IEEE

[50] K. Ni et al., Critical role of interlayer in Hf_0.5_Zr_0.5_O_2_ ferroelectric FET nonvolatile memory performance. IEEE Trans. Electron. Devices. **65**, 2461–2469 (2018). 10.1109/TED.2018.2829122

[51] Q. Zheng et al., Artificial neural network based on doped HfO_2_ ferroelectric capacitors with multilevel characteristics. IEEE Electron. Device Lett. **40**, 1309–1312 (2019). 10.1109/LED.2019.2921737

[52] Y.C. Luo, J. Hur, P. Wang, A.I. Khan, S. Yu, Non-volatile, small-signal capacitance in ferroelectric capacitors. Appl. Phys. Lett. **117**, 0–5 (2020). 10.1063/5.0018937

[53] Y.C. Luo et al., Experimental demonstration of non-volatile capacitive crossbar array for in-memory computing. in. 2021 *IEEE International Electron Devices Meeting (IEDM).* 2021. IEEE. 10.1109/IEDM19574.2021.9720508

[54] S. Mukherjee et al., Capacitive memory window with non-destructive read in ferroelectric capacitors. IEEE Electron. Device Lett. **44**, 1092–1095 (2023). 10.1109/LED.2023.3278599

[55] W. Xu et al., A novel small-signal ferroelectric capacitance-based content addressable memory for area- and energy-efficient lifelong learning. IEEE Electron. Device Lett. **45**, 24–27 (2024). 10.1109/LED.2023.3333849

[56] E. Yu, K.G.K. Saxena, U., K. Roy, Ferroelectric capacitors and field-effect transistors as in-memory computing elements for machine learning workloads. Sci. Rep. **14**, 1–15 (2024). 10.1038/s41598-024-59298-8 38658597 10.1038/s41598-024-59298-8 PMC11551200

[57] Z. Zhou et al., A metal-insulator-semiconductor non-volatile programmable capacitor based on a HfAlO ferroelectric film. IEEE Electron. Device Lett. **41**, 1837–1840 (2020). 10.1109/LED.2020.3035276

[58] Z. Zhou et al., Experimental demonstration of an inversion-type ferroelectric capacitive memory and its 1 kbit crossbar array featuring high C_HCS_/C_LCS_, fast speed, and long retention. in *2022 IEEE Symposium on VLSI Technology (VLSI)*. 2022. IEEE. 10.1109/VLSITechnologyandCir46769.2022.9830291

[59] Z. Zhou et al., Inversion-type ferroelectric capacitive memory and its 1-kbit crossbar array. IEEE Trans. Electron. Devices. **70**, 1641–1647 (2023). 10.1109/TED.2023.3243556

[60] Z. Zhou et al., Non-destructive-read 1T1C ferroelectric capacitive memory cell with BEOL 3D monolithically integrated IGZO access transistor for 4F^2^ high-density integration. in *2023 IEEE Symposium on VLSI Technology (VLSI)*. 2023. IEEE. 10.23919/VLSITechnologyandCir57934.2023.10185243

[61] N. Liu et al., HfO_2_-based ferroelectric optoelectronic memcapacitors. IEEE Electron. Device Lett. **44**, 524–527 (2023). 10.1109/LED.2023.3235909

[62] N. Liu et al., Photoelectric in-memory logic and computing achieved in HfO_2_-based ferroelectric optoelectronic memcapacitors. IEEE Electron. Device Lett. **45**, 1357–1360 (2024). 10.1109/LED.2024.3400990

[63] T.H. Kim et al., Tunable non-volatile gate-to-source/drain capacitance of FeFET for capacitive synapse. IEEE Electron. Device Lett. **44**, 1628–1631 (2023). 10.1109/LED.2023.3311344

[64] O. Phadke, H. Mulaosmanovic, S. Dunkel, S. Beyer, S. Yu, Reliability assessment of ferroelectric nvCAP for small-signal capacitive read-out. in 2024 IEEE International Reliability Physics Symposium (IRPS). 2024. IEEE. 10.1109/IRPS48228.2024.10529440

[65] S. Yu, Y.-C. Luo, T.-H. Kim, O. Phadke, Nonvolatile capacitive synapse: Device candidates for charge domain compute-in-memory. IEEE Electron. Devices Mag. **1**, 23–32 (2023). 10.1109/MED.2023.3293060

[66] O. Phadke, T.-H. Kim, Y.-C. Luo, S. Yu, Ferroelectric nonvolatile capacitive synapse for charge domain compute-in-memory. ECS Trans. **113**, 3–13 (2024). 10.1149/MA2024-01573008mtgabs

[67] I. Yeo, W. He, Y.C. Luo, S. Yu, J.S. Seo, A dynamic power-only compute-in-memory macro with power-of-two nonlinear SAR ADC for nonvolatile ferroelectric capacitive crossbar array. IEEE Solid-State Circuits Lett. **7**, 70–73 (2024). 10.1109/LSSC.2024.3361011

[68] S. Mukherjee et al., Pulse-based capacitive memory window with high non-destructive read endurance in fully BEOL compatible ferroelectric capacitors. in. 2023 *IEEE International Electron Devices Meeting (IEDM).* 2023. IEEE. 10.1109/IEDM45741.2023.10413879

[69] Y.C. Luo, J. Read, A. Lu, S. Yu, A cross-layer framework for design space and variation analysis of non-volatile ferroelectric capacitor-based compute-in-memory accelerators. in Asia and South Pacific Design Automation Conference (ASP-DAC). 2024. 10.1109/ASP-DAC58780.2024.10473887

[70] S.A. Thomas, S. Kushwaha, R. Sharma, D.M. Das, Design and analysis of 3D integrated folded ferro-capacitive crossbar array (FC^2^A) for brain-inspired computing system. IEEE J. Emerg. Sel. Top. Circuits Syst. **14**, 563–574 (2024). 10.1109/JETCAS.2024.3432458

[71] S. Kim, J. Zhou, W.D. Lu, Crossbar RRAM arrays: Selector device requirements during write operation. IEEE Trans. Electron. Devices. **61**, 2820–2826 (2014). 10.1109/TED.2014.2327514

[72] J. Zhou, K.H. Kim, W. Lu, Crossbar RRAM arrays: Selector device requirements during read operation. IEEE Trans. Electron. Devices. **61**, 1369–1376 (2014). 10.1109/TED.2014.2310200

[73] A. Dudley, *Buck. Ferroelectrics for digital information storage and switching* (MIT Digital Computer Laboratory, 1952)

[74] J. Zhou et al., Al-doped and deposition temperature-engineered HfO_2_ near morphotropic phase boundary with record dielectric permittivity (68). in. 2021 *IEEE International Electron Devices Meeting (IEDM).* 2021. IEEE. 10.1109/IEDM19574.2021.9720632

[75] J. Zhou et al., Temperature dependence of ferroelectricity in Al-doped HfO_2_ featuring a high Pr of 23.7 μC/cm^2^. IEEE Trans. Electron. Devices. **67**, 5633–5638 (2020). 10.1109/TED.2020.3032350

[76] L. Jiao et al., First BEOL-compatible IGZO ferroelectric-modulated diode with drastically enhanced memory window: Experiment, modeling, and deep understanding. in. 2023 *IEEE International Electron Devices Meeting (IEDM).* 2023. IEEE. 10.1109/IEDM45741.2023.10413793

[77] Z. Zheng et al., BEOL-compatible MFMIS ferroelectric/anti-ferroelectric FETs - Part II: Mechanism with load line analysis and scaling strategy. IEEE Trans. Electron. Devices. **71**, 5325–5331 (2024). 10.1109/TED.2024.3421184

[78] Z. Lin et al., High-performance BEOL-compatible atomic-layer-deposited In_2_O_3_ Fe-FETs enabled by channel length scaling down to 7 nm: Achieving performance enhancement with large memory window of 2.2 V, long retention > 10 years and high endurance > 10^8^ cycles. in. 2021 *IEEE International Electron Devices Meeting (IEDM).* 2021. IEEE. 10.1109/IEDM19574.2021.9720652

[79] D. Kleimaier et al., Demonstration of a p-type ferroelectric FET with immediate read-after-write capability. IEEE Electron. Device Lett. **42**, 1774–1777 (2021). 10.1109/LED.2021.3118645

[80] M. Hoffmann et al., Fast read-after-write and depolarization fields in high endurance n-type ferroelectric FETs. IEEE Electron. Device Lett. **43**, 717–720 (2022). 10.1109/LED.2022.3163354

[81] J. Li, M. Si, Y. Qu, X. Lyu, P.D. Ye, Quantitative characterization of ferroelectric/dielectric interface traps by pulse measurements. IEEE Trans. Electron. Devices. **68**, 1214–1220 (2021). 10.1109/TED.2021.3053497

[82] Y. Xiang et al., Physical insights on steep slope FEFETs including nucleation-propagation and charge trapping. in. 2019 *IEEE International Electron Devices Meeting (IEDM).* 2019. IEEE. 10.1109/IEDM19573.2019.8993492

[83] Y. Xiang et al., Implication of channel percolation in ferroelectric FETs for threshold voltage shift modeling. in. 2020 *IEEE International Electron Devices Meeting (IEDM).* 2020. IEEE. 10.1109/IEDM13553.2020.9371907

[84] T. Ali et al., High endurance ferroelectric hafnium oxide-based FeFET memory without retention penalty. IEEE Trans. Electron. Devices. **65**, 3769–3774 (2018). 10.1109/TED.2018.2856818

[85] K. Toprasertpong et al., Improved ferroelectric/semiconductor interface properties in Hf_0.5_Zr_0.5_O_2_ ferroelectric FETs by low-temperature annealing. IEEE Electron. Device Lett. **41**, 1588–1591 (2020). 10.1109/LED.2020.3019265

[86] S. Oh et al., Improved endurance of HfO_2_-based metal-ferroelectric-insulator-silicon structure by high-pressure hydrogen annealing. IEEE Electron. Device Lett. **40**, 1092–1095 (2019). 10.1109/LED.2019.2914700

[87] Y. Chen et al., Improved TDDB reliability and interface states in 5-nm Hf_0.5_Zr_0.5_O_2_ ferroelectric technologies using NH_3_ plasma and microwave annealing. IEEE Trans. Electron. Devices. **67**, 1295–1298 (2021). 10.1109/TED.2020.2973652

[88] M. Nguyen et al., Wakeup-free and endurance-robust ferroelectric field-effect transistor memory using high pressure annealing. IEEE Electron. Device Lett. **42**, 1295–1298 (2021). 10.1109/LED.2021.3096248

[89] A.J. Tan et al., A nitrided interfacial oxide for interface state improvement in hafnium zirconium oxide-based ferroelectric transistor technology. IEEE Electron. Device Lett. **39**, 95–98 (2018). 10.1109/LED.2017.2772791

[90] L. Zhang et al., Ultrathin metal/amorphous-silicon/metal diode for bipolar RRAM selector applications. IEEE Electron. Device Lett. **35**, 199–201 (2014). 10.1109/LED.2013.2293591

[91] D. Kau et al., A stackable cross point phase change memory. in *2009 IEEE International Electron Devices Meeting (IEDM).* 2009. IEEE. 10.1109/IEDM.2009.5424263

[92] W. Lee et al., Varistor-type bidirectional switch (J_MAX_>10^7^A/cm^2^, selectivity∼10^4^) for 3D bipolar resistive memory arrays. in *2012 IEEE Symposium on VLSI Technology (VLSI)*. 2012. IEEE. 10.1109/VLSIT.2012.6242449

[93] K.U. Demasius, A. Kirschen, S. Parkin, Energy-efficient memcapacitor devices for neuromorphic computing. Nat. Electron. **4**, 748–756 (2021). 10.1038/s41928-021-00649-y

